# Detailed analysis of metastatic colorectal cancer patients who developed cardiotoxicity on another fluoropyrimidine and switched to S-1 treatment (subgroup analysis of the CardioSwitch-study)

**DOI:** 10.2340/1651-226X.2024.24023

**Published:** 2024-05-02

**Authors:** Sampsa Kinos, Helga Hagman, Päivi Halonen, Leena-Maija Soveri, Mary O’Reilly, Per Pfeiffer, Jan-Erik Frödin, Halfdan Sorbye, Eetu Heervä, Gabor Liposits, Raija Kallio, Annika Ålgars, Raija Ristamäki, Tapio Salminen, Maarit Bärlund, Carl-Henrik Shah, Ray McDermott, Rebecka Röckert, Petra Flygare, Johannes Kwakman, Arco J. Teske, Cornelis Punt, Bengt Glimelius, Pia Österlund

**Affiliations:** a Department of Oncology, Tays Cancer Center, Tampere University Hospital, Tampere, Finland; b Faculty of Medicine and Health Technology, University of Tampere; c Department of Oncology, Skåne University Hospital, Malmö, Sweden; d Department of Oncology, Helsinki University Hospital and University of Helsinki, Helsinki, Finland; e Department of Oncology, St Vincent’s University Hospital and University College Dublin, Dublin, Ireland; f Department of Oncology, Odense University Hospital, Odense, Denmark; g Department of Oncology, Karolinska University Hospital, Stockholm, Sweden; h Department of Oncology, Haukeland University Hospital, Bergen, Norway; i Department of Oncology, Turku University Hospital and University of Turku, Turku, Finland; j Department of Oncology, Regional Hospital West Jutland, Hjørring, Denmark; k Department of Oncology, Oulu University and University Hospital, Oulu, Finland; l Department of Oncology, Uppsala University, Uppsala, Sweden; m Department of Oncology, Sundsvall Hospital, Sundsvall, Sweden; n Department of Medical Oncology, University Medical Centre Utrecht, Utrecht University, Utrecht, the Netherlands; o Department of Cardiology, University Medical Centre, Utrecht University, Utrecht, The Netherlands; p Depatment of Epidemiology, Jules Center for Health Sciences and Primary Care, University Medical Center Utrecht, Utrecht University, Utrecht, The Netherlands; q Department of Oncology and Pathology, Karolinska Institutet, Stockholm, Sweden; r Tema Cancer, Department of GI-cancer, Karolinska University Hospital, Stockholm, Sweden

**Keywords:** Metastatic colorectal cancer, fluoropyrimidines, S-1, cardiotoxicity, capecitabine, 5-fluorouracil, metastasectomy

## Abstract

**Background and purpose:**

The CardioSwitch-study demonstrated that patients with solid tumors who develop cardiotoxicity on capecitabine or 5-fluorouracil (5-FU) treatment can be safely switched to S-1, an alternative fluoropyrimidine (FP). In light of the European Medicines Agency approval of S-1 in metastatic colorectal cancer (mCRC), this analysis provides more detailed safety and efficacy information, and data regarding metastasectomy and/or local ablative therapy (LAT), on the mCRC patients from the original study.

**Materials and methods:**

This retrospective cohort study was conducted at 12 European centers. The primary endpoint was recurrence of cardiotoxicity after switch. For this analysis, safety data are reported for 78 mCRC patients from the CardioSwitch cohort (*N* = 200). Detailed efficacy and outcomes data were available for 66 mCRC patients.

**Results:**

Data for the safety of S-1 in mCRC patients were similar to the original CardioSwitch cohort and that expected for FP-based treatment, with no new concerns. Recurrent cardiotoxicity (all grade 1) with S-1-based treatment occurred in 4/78 (5%) mCRC patients; all were able to complete FP treatment. Median progression-free survival from initiation of S-1-based treatment was 9.0 months and median overall survival 26.7 months. Metastasectomy and/or LAT was performed in 33/66 (50%) patients, and S-1 was successfully used in recommended neoadjuvant/conversion or adjuvant-like combination regimens and schedules as for standard FPs.

**Interpretation:**

S-1 is a safe and effective FP alternative when mCRC patients are forced to discontinue 5-FU or capecitabine due to cardiotoxicity and can be safely used in the standard recommended regimens, settings, and schedules.

## Introduction

Fluoropyrimidine (FP) chemotherapy regimens based on intravenous (i.v.) 5-fluorouracil (5-FU) or oral capecitabine (CAP) are the backbone of recommended therapy for metastatic colorectal cancer (mCRC) [1]. However, cardiotoxicity is a serious, potentially fatal, side effect of 5-FU or CAP treatment that is difficult to manage and is observed in approximately 4%–6% of CRC patients [2–8].

The indications for FP treatment in the metastatic setting may be of palliative or curative intent. In the palliative setting, the aim is prolonged survival with good quality of life, often achieved by de-intensification of treatment. When curative-intent is the goal, FP is used as neoadjuvant or conversion treatment before metastasectomy or local ablative therapy (LAT), and/or in the adjuvant-like setting after metastasectomy. In all of these settings, FPs are the cornerstone for combination chemotherapy (combined with a biologic for conversion therapy) [1].

S-1, a combination of tegafur with two metabolic inhibitors designed to slow the metabolism and improve the side-effect profile, is an alternative FP associated with lower rates of cardiotoxicity [9–12]. The CardioSwitch-study demonstrated that patients with solid tumors who develop cardiotoxicity on CAP or 5-FU can be safely switched to S-1 and continue with guideline-recommended FP treatment [13]. Based on the positive risk/benefit analysis demonstrated by the CardioSwitch-study and other studies [12–16], S-1 was approved by the European Medicines Agency (EMA) for use ‘as monotherapy or in combination with oxaliplatin or irinotecan, with or without bevacizumab, in patients with mCRC for whom it is not possible to continue treatment with another FP due to hand-foot syndrome (HFS) or cardiovascular toxicity that developed in the adjuvant or metastatic setting’ [17, 18]. As a result, S-1 has now been added to the most recent European mCRC treatment guideline as an alternative to i.v. 5-FU- or CAP-based chemotherapy in the case of cardiotoxicity and/or HFS [1].

As part of the approval process, summarized safety and efficacy data for the mCRC cohort were shared with EMA to allow assessment of benefit versus risk in this indication. Some of the data for this ‘SmPC cohort’ (described in more detail in the methods section) were included in the Summary of Product Characteristics (SmPC) for S-1 (Teysuno) [17, 18]. However, detailed safety and efficacy data for all of the mCRC patients in the study were not part of the original publication and are relevant for clinicians to allow them to make informed treatment decisions with regard to switching their mCRC patients to S-1-based [13]. In addition, data on outcomes for mCRC patients who were switched to S-1 before or after resection or ablation of metastases are now available and can assist in clinical decision making.

The objectives of this analysis were to evaluate the safety and efficacy data from the mCRC subgroup of the CardioSwitch-study in detail and to provide further information with regard to outcomes associated with metastasectomy and/or LAT in the context of S-1 regimens.

## Materials and methods

### Study design and patients

The CardioSwitch-study [13] was a multicenter, retrospective cohort study conducted at 13 centers in Europe. The study was approved by the local ethics committee at each institution, if required, and conducted according to Good Clinical Practice Guidelines and the Declaration of Helsinki, as applicable for registry studies. Detailed information regarding the participating centers and investigators, as well as the study protocol have been previously published [13].

The primary endpoint for the study was recurrence of cardiotoxicity after switch to S-1-based treatment from any other FP due to cardiotoxicity. Secondary endpoints included cardiac symptoms and diagnostic work-up, timeline of cardiotoxicity, dose intensity, safety, and outcomes of treatment, including metastasectomy and/or LAT.

Patients included in the original solid tumor cohort (*N* = 200) have been described [13]. Included mCRC (*n* = 78) patients were from 12 centers in 6 countries (Finland, Sweden, Denmark, Norway, Ireland, and the Netherlands). All patients had experienced a cardiotoxic adverse event (AE) on FP-based treatment (oral CAP or 5-FU as bolus or de Gramont infused regimen) and had metastatic disease at the time they were switched to S-1-based treatment.

Data are presented here for three cohorts of mCRC patients, all of whom were included in the CardioSwitch-study [13] as follows:

- A graphical depiction of these cohorts is shown in Supplementary Figure 1
- The ‘Safety cohort’ includes all 78 patients who received treatment with S-1 for metastatic disease. This includes patients who had mCRC at baseline and were switched to S-1 after development of cardiotoxicity on initial FP treatment (*n* = 65) and patients who developed mCRC after initial cardiotoxicity in the adjuvant setting and were then treated with S-1 (*n* = 13).
- The ‘Efficacy cohort’ includes 66 mCRC patients for whom efficacy data were available. Efficacy data were not available for 12 Dutch patients who had mCRC at baseline (mCRC data cannot be retrieved as they were anonymized at data collection). The Efficacy cohort includes all patients who had mCRC at baseline for whom efficacy data were available (*n* = 53) as well as the 13 patients who had cardiotoxicity during adjuvant treatment (e.g., initial FP-induced cardiotoxicity, but then received S-1 for metastatic disease). Treatment data are updated here for this Efficacy cohort.
- The ‘SmPC cohort’ includes the 53 patients with mCRC at baseline for whom efficacy information were available and who were included in data provided for the Teysuno SmPC [18].

### Treatment of mCRC patients

The FP-based regimens that caused initial cardiotoxicity were CAP monotherapy; CAP plus oxaliplatin (CAPOX); CAP plus irinotecan (CAPIRI); leucovorin plus Nordic bolus 5-FU (FLv); leucovorin plus i.v. bolus then infusional 5-FU (i.e., de Gramont/LV5FU2); leucovorin, de Gramont 5-FU, plus oxaliplatin (FOLFOX); leucovorin, de Gramont 5-FU, plus irinotecan (FOLFIRI); leucovorin, bolus 5-FU, plus oxaliplatin (FLOX); or leucovorin, bolus 5-FU plus irinotecan (FLIRI) [13, 19–22]. S-1-based regimens used were S-1 monotherapy; S-1 plus oxaliplatin (SOX), or S-1 plus irinotecan (IRIS) [13]. Biologic drugs added were bevacizumab, aflibercept, panitumumab, or cetuximab. Detailed information on dosing for S-1-based chemotherapy with or without biologic drugs is available in Supplementary Table 1 in Osterlund et al. [13].

### Cardiotoxicity definition

As part of the original study cohort, cardiac AEs in patients with mCRC were defined and graded using the Cardiac Disorders in National Institutes of Health Common Terminology Criteria for Adverse Events 4.0 criteria and causality to FPs was assessed according to World Health Organization Uppsala Monitoring Center guidelines [13]. Based on clinical records, two experienced oncologists graded cardiac disorders and determined causality, with consensus reached for all patients.

### Treatment definitions

Curative-intent treatments are presented as Neoadjuvant/Conversion or Adjuvant/Adjuvant-like. Conversion therapy, often combination chemotherapy with biologics, is given with the aim of converting unresectable metastases to resectable [1]. Neoadjuvant therapy refers to chemotherapy administered before planned surgical intervention to improve outcome by destroying micrometastases. Adjuvant therapy is administered after curative-intent surgery of the primary tumor and adjuvant-like therapy after curative metastasectomy and/or LAT. Curative treatment means eradication of all tumors with R0-resection or A0-ablation [1]. Palliative chemotherapy was defined as first-, second-, and third or later-line treatment.

### Statistical analyses

Median and range are reported for continuous variables. The Kaplan-Meier estimate was used for survival analyses, and Cox regression for comparison of survival between metastasectomy/LAT or systemic therapy only patients. Proportional hazard assumption was assessed by plotting Schoenfeld residuals and by inspection of the Kaplan-Meier plots. Overall survival (OS) and progression-free survival (PFS) were determined from the initiation of S-1-based treatment for metastatic disease and OS was also determined from diagnosis of mCRC. The endpoint was death from any cause or censored at last date of follow-up. Data cut-off was 13th July, 2023 for the updated population of 66 mCRC patients.

## Results

### Patients

Baseline characteristics for the Safety cohort (*N* = 78) are shown in Table 1, and baseline characteristics for the Efficacy cohort (*n* = 66) and the SmPC cohort (*n* = 53) in Supplementary Table 1. The median age (range) of patients included in the Safety cohort was 68 (19–85) years, 60% were male, and 50% had one or more cardiovascular comorbidity at baseline (details for comorbidities in Supplementary Table 2).

**Table 1:** Baseline characteristics of mCRC patients included in the Safety cohort.

		Total mCRC, *N* = 78*n* (%)	
Age, years,	Median (range)	68 (19–85)	
Sex	Male	47 (60)	
ECOG	0–1	59 (76)	
	2	8 (10)	
	Not available	11 (14)	
Cardiovascular comorbidity	Yes	39 (50)	
	No	39 (50)	
Primary tumor location	Right colon	19 (29)	
	Left colon	24 (36)	
	Rectum	14 (21)	
	Colon unspecified/multiple	9 (14)	
Primary tumor resected	Yes	49 (63)	
	No	26 (33)	
	Not available	3 (4)	
Pelvic radiotherapy	Yes	4 (5)	
	No	70 (90)	
	Not available	4 (5)	
		Initial FP causing cardiotoxicity, *N* = 78 *n* (%)	Switch to S-1 *N* = 78 *n* (%)
Treatment intent	Adjuvant/Adjuvant-like	21 (27)	12 (15)
	Neo-adjuvant/Conversion	19 (24)	17 (22)
	1st line	31 (40)	38 (49)
	2nd line	4 (5)	6 (8)
	3rd or later line	3 (4)	5 (6)
Fluoropyrimidine	Capecitabine	60 (77)	-
	Bolus/infused/de Gramont 5-FU	13 (17)	-
	Nordic bolus 5-FU	5 (6)	-
	S-1	-	78 (100)
Combined drugs	Single fluoropyrimidine	27 (35)	29 (37)
	Oxaliplatin*	48 (62)	34 (44)
	Irinotecan*	5 (6)	13 (17)
	Other cytotoxic**	-	2 (3)
	Bevacizumab	29 (37)	29 (37)
	Cetuximab	2 (3)	1 (1)
	Concurrent radiotherapy	1 (1)	2 (3)

5-FU: 5-fluorouracil; ECOG: Eastern Cooperative Oncology Group; FP: fluoropyrimidine; mCRC: metastatic colorectal cancer.

* Two patients received both oxaliplatin and irinotecan, one patient received alternating 5-FU, leucovorin, plus oxaliplatin (FOLFOX) and 5-FU, leucovorin, plus irinotecan (FOLFIRI) within the Nordic-8 study and one patient received 5-FU, leucovorin, oxaliplatin, and irinotecan (FOLFOXIRI).

** carboplatin and temozolomide.

### Initial cardiotoxicity on capecitabine- or 5-FU-based treatment

Initial FP treatment regimens were CAP-based (CAP, CAPOX, or CAPIRI) in 77% of patients, 17% received 5-FU as the de Gramont regimen (LV5FU2, mFOLFOX, or FOLFIRI), and 6% as Nordic bolus (FLv, FLOX, or FLIRI; Table 1). FP monotherapy was administered to 35% patients. Combination regimens included oxaliplatin in 62%, irinotecan in 6%, bevacizumab in 37%, cetuximab in 3%, and radiotherapy in 1%. The treatment intention was neoadjuvant/conversion in 24%, adjuvant/adjuvant-like in 27%, and palliative in 49%.

Details of the cardiotoxicity with CAP- or 5-FU-based treatment in the Safety cohort are shown in Table 2. Most cardiotoxicity occurred in the first (71%) or second cycle of treatment (13%) with a median time (range) from FP treatment onset of 5 (0–466) days. The most common cardiac symptoms were chest pain (62%) and acute coronary syndrome (37%). Less frequent atrial fibrillation (5%), tachycardia (5%), heart failure (3%), bradycardia (1%), and prolonged QT interval (1%) were reported. Grade 3 or 4 cardiotoxicity was experienced by 55%. Initial FP treatment was permanently discontinued in 95% of the patients and 92% of the cardiotoxicity reactions were considered to be either probably related (62%) or related (30%) to the FP treatment. Details of cardiotoxicity for the SmPC cohort and the Efficacy cohort are presented in Supplementary Table 3.

**Table 2:** Cardiotoxicity and other adverse events during initial fluoropyrimidine treatment and S-1-based treatment, Safety cohort.

		Initial FP causing cardiotoxicity, *N* = 78 *n* (%)	Switch to S-1-based, *N* = 78 *n* (%)
Recurrent cardiotoxicity	No	Not applicable	74 (95)
	Yes	Not applicable	4 (5)
Number of cycles to cardiotoxicity	1	55 (71)	1 (1)
	2	10 (13)	1 (1)
	3	5 (6)	-
	4 to 14	8 (10)	2 (3)
Multiple cardiotoxic symptoms	No	67 (86)	4 (5)
	Yes	11 (14)	-
Cardiotoxicity symptoms*	Chest pain	48 (62)	3 (4)
	Acute coronary syndrome	29 (37)	-
	Atrial fibrillation	4 (5)	-
	Heart failure	2 (3)	-
	Arrythmia	5 (6)	1 (1)
	Prolonged QT interval	1 (1)	-
Worst cardiotoxicity grade	1	8 (10)	4 (5)
	2	27 (35)	-
	3	36 (46)	-
	4	7 (9)	-
Action with fluoropyrimidine	None	-	1 (1)
	Dose delayed	-	1 (1)
	Dose reduced	-	1 (1)
	Temporarily discontinued	4 (5)	1 (1)
	Permanently discontinued	74 (95)	
Recovery from cardiac event	With sequelae	1 (4)	-
	Without sequelae	27 (96)	4 (5)
Causality	Possibly related	7 (9)	-
	Probably related	48 (62)	2 (3)
	Related	23 (30)	-
	Not related	-	2 (3)
Non-cardiac adverse events**	Neutropenia	1 (1)	10 (13)
	Anemia	-	1 (1)
	Stomatitis	1 (1)	2 (3)
	Diarrhea	2 (3)	8 (10)
	Nausea	1 (1)	3 (4)
	Infection	1 (1)	6 (8)
	Neuropathy	5 (6)	10 (13)
	Hand-foot syndrome	3 (4)	1 (1)
	Thromboembolism	-	4 (5)
	Fatigue	-	1 (1)
	Pneumonitis	-	1 (1)
	Rash	-	1 (1)
	Any non-hematologic event	11 (14)	23 (29)

FP: fluoropyrimidine.

* Eleven patients experienced more than one cardiac symptom.

** Hematologic toxicities grade 3–4, others grade 2–4.

Non-cardiac AEs related to initial FP treatment, which lasted two cycles or less in 84% of patients, are shown in Table 2. The most common non-cardiac AEs related to initial 5-FU- or CAP-based treatment included neuropathy (6%), HFS (4%), and diarrhea (3%). Non-hematologic grade 2–4 AEs were experienced by 14% of patients.

### Switch to S-1-based therapy – treatment regimens

A total of 37% of patients in the Safety cohort received S-1 monotherapy upon switch after discontinuation of initial FP due to cardiotoxicity (Table 1). S-1 at the time of switch was combined with oxaliplatin (44%), irinotecan (17%), or bevacizumab (37%). During any line of treatment with S-1, oxaliplatin was combined in 60%, irinotecan in 49%, bevacizumab in 45%, aflibercept in 1%, panitumumab in 4%, and cetuximab in 3%. The median number of cycles in any line for the most common combinations are presented in Table 3. Of note, patients were able to stay on IRIS for median of seven cycles (range 1–41).

**Table 3:** Clinically significant* adverse events on S-1-based treatment according to treatment regimen, more than one line present per patient (Safety cohort, *N* = 78)

	S-1 monotherapy** *n* = 42 (%)	SOX^†^ *n* = 47 (%)	IRIS^‡^ *n* = 38 (%)
Number of cycles, median (range)	4 (1–31)	6 (1–25)	7 (1–41)
Anemia	-	-	1 (3)
Neutropenia	1 (2)	2 (5)	5 (13)
Thrombocytopenia	-	1 (2)	-
Stomatitis	1 (2)	-	1 (3)
Diarrhea	1 (2)	1 (2)	4 (11)
Nausea	-	-	2 (5)
Infection	3 (7)	1 (2)	2 (5)
Neuropathy	-	7 (17)	-
Hand-foot syndrome	1 (2)	-	-
Thromboembolism	-	1 (2)	1 (3)
Rash	-	-	1 (3)
Lung toxicity	-	-	1 (3)
Fatigue	-	-	1 (3)

SOX: S-1 plus oxaliplatin; IRIS: S-1 plus irinotecan.

* Clinically significant defined as Grade 2–4, except for hematological for which grade 3–4 are shown.

** ± bevacizumab.

† S-1 plus oxaliplatin ± bevacizumab.

‡ S-1 plus irinotecan ± bevacizumab.

In the Efficacy cohort (updated data not available for 12 anonymized patients) vascular endothelial growth factor (VEGF) inhibitors were combined with chemotherapy in 53% (bevacizumab median 7 [range 1–30] cycles, aflibercept in one patient for 13 cycles). EGFR inhibitors were added in 8% (median 2 [range 1–9] cycles, of whom 3 received panitumumab and 2 cetuximab).

### Switch to S-1-based therapy – efficacy

In the Efficacy cohort (*n* = 66), median PFS (mPFS) from initiation of S-1-based treatment for metastatic disease was 9.0 months and median OS (mOS) was 26.7 months.

**Figure 1:**
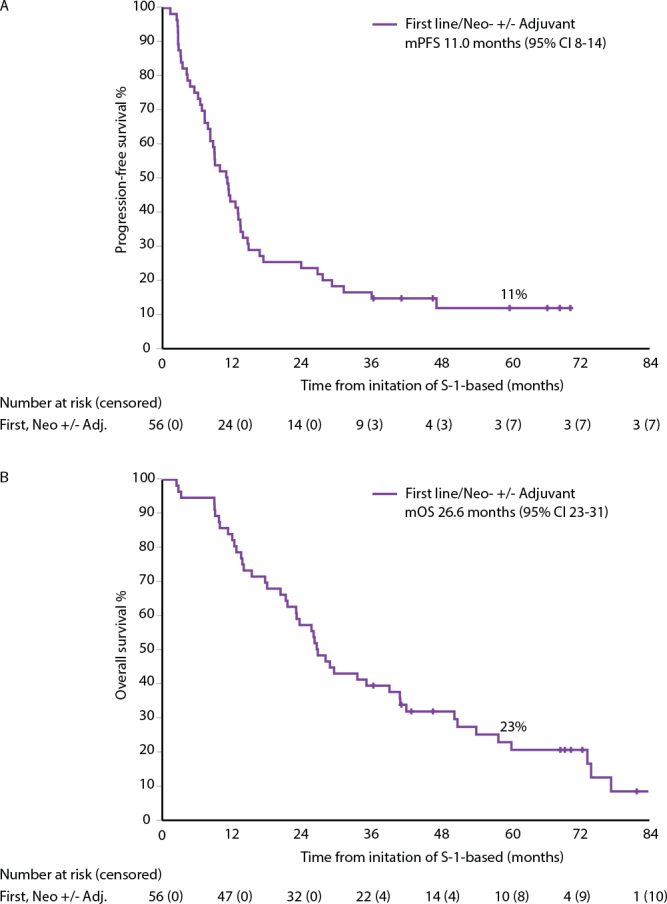
Progression-free (PFS, panel A) and overall survival (OS, panel B) for patients receiving S-1-based treatment for metastatic disease as first-line (neoadjuvant/conversion and/or adjuvant-like after metastasectomy; *n* = 56) in Efficacy cohort.

The subgroup of patients treated in first-line, with a palliative intent in 27 and a curative intent in 29 (with neoadjuvant/conversion [*n* = 18], or adjuvant-like after metastasectomy [*n* = 11]) had a mPFS of 11.0 months and mOS of 26.6 months (Figure 1, Supplementary Table 1). Corresponding 5-year PFS rate was 11% and OS rate was 23%.

Patients on palliative S-1 treatment as later-line treatment (second-line *n* = 6, third- or later-line *n* = 4) had mPFS of 2.8 months and mOS of 12.7 months.

### Switch to S-1-based therapy – recurrent cardiotoxicity and safety

Among the 78 mCRC patients in the Safety cohort, 5% (*n* = 4) experienced recurrent cardiotoxicity on S-1-based treatment; similar to the rate in the entire CardioSwitch cohort (4%) (Table 2) [13]. These four patients (three female) were of 57–70 years, ECOG 0-1, and two of them had no cardiovascular comorbidities. Time-to-recurrence of cardiotoxicity on S-1-based therapy was 7, 22, 95, and 195 days. Three of these patients had experienced grade 3 acute coronary syndrome as their initial cardiotoxicity on the original FP. The recurrent cardiotoxicities on S-1-based therapy were grade 1 chest pain in three and grade 1 tachycardia in one (Table 2). All patients were able to continue treatment until progression or completion of adjuvant-like treatment with S-1-based therapy.

All four patients who experienced recurrence on S-1-based therapy had received combination therapy including oxaliplatin as initial FP-based treatment, and continued with S-1 as monotherapy (*n* = 2) or as SOX ± bevacizumab (*n* = 2; details in Supplementary Table 7 in Osterlund et al., 2022) [13].

The most common non-cardiac AEs in the Safety cohort while on S-1-based therapy were neuropathy (13%), neutropenia (13%), diarrhea (10%), infection (8%), and thromboembolism (5%), and 29% experienced non-hematologic grade 2–4 AEs (Table 2). The AEs for the SmPC cohort are presented in Supplementary Table 4.

Table 3 shows clinically significant AEs according to S-1 regimen for patients in the Safety cohort. The most common AE for S-1 monotherapy (± bevacizumab) was infection (7%), while neuropathy was observed in 17% of patients who received S-1 in combination with oxaliplatin (SOX) (± bevacizumab), and neutropenia was observed in 13% of patients who received S-1 in combination with irinotecan (IRIS) (± bevacizumab). Diarrhea was reported in 2% of patients on S-1 monotherapy, 2% of those who received SOX, and 11% of those who received IRIS.

### Impact of S-1 therapy on metastasectomy and local ablative therapy outcomes

Detailed data on metastasectomy and/or LAT (*n* = 33) were available for the Efficacy cohort of 66 patients. Metastasectomy was performed in 31 patients (47%) and ablation in 5 patients (8%, of whom 3 underwent both metastasectomy and LAT) (Table 4). In total, 33 patients (50%) had a mean of 1.6 procedures per patient (Table 5). Of these 33 with metastasectomy/LAT, 13 patients had received S-1 based before metastasectomy, 8 both before and after switch, i.e., repeated metastasectomies performed, 9 after switch.

**Table 4:** Baseline and treatment information for patients who received systemic therapy only or metastasectomy and/or local ablative therapy (LAT) in any line of treatment for the Efficacy cohort (*n* = 66).

		Systemic therapy only *n* (%)	Metastasectomy ± LAT before switch *n* (%)	Metastasectomy ± LAT before and after switch *n* (%)	Metastasectomy ± LAT after switch *n* (%)
		33 (50)	12 (18)	12 (18)	9 (14)
Sex	Female	14 (42)	6 (50)	2 (17)	4 (44)
	Male	19 (58)	6 (50)	10 (83)	5 (56)
ECOG performance status	0	3 (12)	3 (30)	6 (60)	3 (38)
	1	18 (72)	6 (60)	4 (40)	5 (63)
	2	4 (16)	1 (10)	0 (0)	0 (0)
Primary tumor location	Right colon	9 (27)	5 (42)	3 (25)	2 (22)
	Left colon	13 (39)	1 (8)	6 (50)	4 (44)
	Rectum	8 (24)	3 (25)	2 (17)	1 (11)
	Unknown	3 (9)	3 (25)	1 (8)	2 (22)
Surgery of primary	No	14 (42)	1 (8)	3 (25)	2 (22)
	Yes	19 (58)	11 (92)	9 (75)	7 (78)
Radiotherapy for primary	No	31 (94)	10 (83)	12 (100)	9 (100)
	Yes	2 (6)	2 (17)	0 (0)	0 (0)
	No	13 (39)	6 (50)	3 (25)	2 (22)
Single S-1	*± biologic	17 (52)	5 (42)	3 (25)	2 (22)
S-1 + oxaliplatin (SOX)	^†^± biologic	16 (49)	7 (58)	9 (75)	7 (78)
S-1 + irinotecan (IRIS)	^‡^± biologic	17 (52)	7 (58)	7 (58)	6 (67)
Bevacizumab	With chemotherapy	20 (61)	3 (25)	6 (50)	6 (67)
EGFR-inhibitor	With chemotherapy	3 (9)	0 (0)	1 (8)	1 (11)

ECOG: Eastern Cooperative Oncology Group; EGFR: epidermal growth factor receptor; LAT: local ablative therapy.

* Biologic: bevacizumab in 25, EGFR-inhibitor in 4.

† Biologic: bevacizumab in 22, EGFR-inhibitor in 2.

‡ Biologic: bevacizumab in 22, EGFR-inhibitor in 4, aflibercept in 1.

**Table 5:** Description of metastasectomy and/or local ablative therapy (LAT) procedures according to time of switch.

		Metastasectomy ± LAT before switch *n* (%)	Metastasectomy ± LAT before and after switch *n* (%)	Metastasectomy ± LAT after switch *n* (%)
		12 (18)	12 (18)	9 (14)
Number of procedures	1	9 (75)	6 (50)	5 (56)
	2	2 (17)	4 (33)	3 (33)
	3	1 (8)	1 (8)	1 (11)
	6	0 (0)	1 (8)	0 (0)
Procedures	Metastasectomy	11 (92)	9 (75)	8 (89)
	Metastasectomy and LAT	1 (8)	1 (8)	1 (11)
	LAT	0 (0)	2 (17)	0 (0)
Liver procedures	Number of patients	3 (25)	9 (75)	8 (89)
	1 liver resection	2 (17)	4 (33)	6 (67)
	2 liver resections	1 (8)	4 (33)	1 (11)
	3 liver resections	0 (0)	0 (0)	1 (11)
	1 thermoablation	1 (8)	1 (8)	1 (11)
Lung procedures	Number of patients	3 (25)	4 (33)	0 (0)
	1 lung resection	2 (67)	3 (25)	0 (0)
	2 lung resections	1 (33)	0 (0)	0 (0)
	3 lung resections	0 (0)	1 (8)	0 (0)
	SBRT lung lesion	0 (0)	1 (8)	0 (0)
Cytoreductive surgery	Number of patients	2 (17)	2 (17)	1 (11)
± HIPEC	1 CRS ± HIPEC	1 (50)	2 (100)	0 (0)
	2 CRS ± HIPEC	1 (50)	0 (0)	1 (100)
Distant lymphadenectomy	Number of patients	3 (25)	0 (0)	0 (0)
	1 procedure	3 (100)	0 (0)	0 (0)
Sub-/Cutaneous extirpation	Number of patients	2 (17)	1 (8)	0 (0)
	1 procedure	2 (100)	1 (100)	0 (0)
	EBRT for subcutaneous mass	0 (0)	1 (100)	0 (0)
Radicality	R0	9 (75)	8 (67)	8 (89)
	R1	2 (17)	1 (8)	0 (0)
	R2	1 (8)	0 (0)	1 (11)
	A0	0 (0)	2 (17)	0 (0)
Complications of procedures	No	7 (58)	5 (42)	4 (44)
	Aspiration pneumonia	0 (0)	1 (8)	0 (0)
	Missing	5 (42)	6 (50)	5 (56)

CRS: cytoreductive surgery; EBRT: external beam radiotherapy; HIPEC: hyperthermic intraperitoneal chemotherapy; LAT: local ablative therapy; SBRT: stereotactic body radiotherapy.

In the neoadjuvant/conversion setting, 95% (20/21) received S-1 in combination with oxaliplatin or irinotecan, with or without biologics (bevacizumab, 57% [12/21] or panitumumab/cetuximab, 10% [2/21]), and 17 had successful metastasectomy/LAT. If metastasectomy and/or LAT was performed before switch to S-1 (*n* = 13) or before and after switch (*n* = 8), S-1 was administered as adjuvant-like treatment in 8 (S-1, SOX, IRIS), as neoadjuvant/conversion before re-metastasectomy/LAT in 5, or after recurrence as palliative chemotherapy in 8 (all as SOX or IRIS, plus bevacizumab in 3).

Liver procedures (liver resection and/or LAT) were performed in 20 (30%) patients (Table 5), and 85% of these procedures were facilitated by S-1 administered as neoadjuvant/conversion therapy. Radical (R0 or A0) procedures were achieved in 85% of patients.

Lung resection or stereotactic body radiotherapy (SBRT) was performed in 8 (12%) patients with curative intent S-1-based therapy as neoadjuvant/conversion therapy in 50% and as adjuvant-like in 40% patients. All procedures were radical.

Cytoreductive surgery was performed in 5 (8%) patients and 40% of these had received S-1-based treatment as neoadjuvant/conversion. Distant lymphadenectomy was performed in three patients, and all of these procedures were performed before switch to S-1-based therapy. Subcutaneous and cutaneous surgery or radiotherapy was performed in three patients, of which one had had neoadjuvant/conversion therapy with S-1-based therapy.

Complications in conjunction with procedures were noticed in 1 patient (6%, information available in 17 out of 33 patients) with aspiration pneumonia after lung resection.

**Figure 2:**
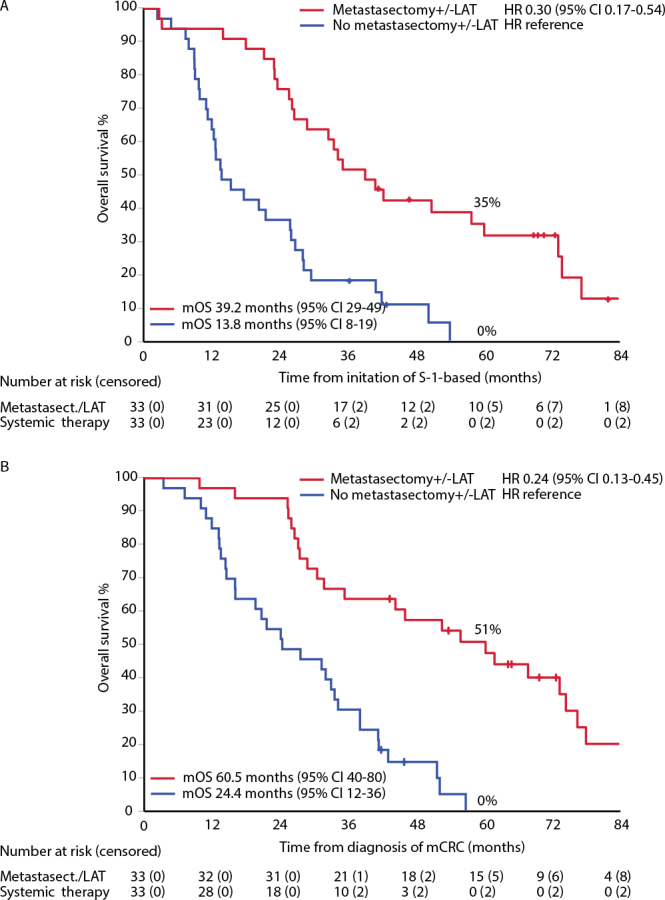
Overall survival from initiation of S-1-based therapy for metastatic colorectal cancer (mCRC, panel A) and from diagnosis of mCRC (panel B) (Efficacy cohort, *n* = 66). Metastasectomy/local ablative therapy (LAT) was performed before switch in 13, before/after in 8 and after in 9 (posing a risk of guarantee-time bias).

OS from initiation of S-1-based therapy for metastatic disease was longer in patients with metastasectomy and/or LAT compared with patients who received only systemic therapy (Figure 2A; HR 0.30; mOS 39 vs. 14 months, and 5-year OS rate 35% vs. 0%). Survival from the date of metastatic disease was longer for patients who had metastasectomy and/or LAT during their treatment trajectory compared with non-surgically treated patients who received systemic treatment only (HR 0.24; Figure 2B). The 5-year OS rates were 51% with a mOS of 61 months for those who underwent a strategy of maximizing surgical and ablative procedures with aggressive S-1-based therapy enabling this approach in 21 (64%) out of 33 patients.

## Discussion

Cardiotoxicity almost always leads to permanent discontinuation of 5-FU or CAP and this presents a difficult challenge since most effective treatment regimens for mCRC are based on an FP backbone [1]. The central finding of this analysis is that all mCRC patients who had experienced cardiotoxicity leading to discontinuation of initial FP treatment were able to safely continue effective treatment with S-1-based regimens. In this mCRC group, only 5% experienced recurrent cardiotoxicity on S-1-based treatment, comparable to the 4% observed in the CardioSwitch cohort also including other solid tumors [13]. A similar low recurrence rate upon switching to S-1-based treatment after FP-induced cardiotoxicity was previously reported in two case series [12, 14]. In addition, the nature of the recurrent cardiotoxicity was less severe (grade 1) compared with 55% having grade 3 or 4 on the initial FP treatment, in line with the larger CardioSwitch-study [13].

No new cardiac risk markers were detected in this analysis. In this group, half of the mCRC patients did not have any pre-existing cardiovascular comorbidities. Thus, predicting FP-induced cardiotoxicity, seen in 4%–6% of mCRC patients, remains a challenge for the clinician [5, 6].

The overall rate of clinically significant AEs with S-1-based treatment was in line with previously published studies, and the spectrum of AEs was consistent with that expected from FP-based treatment. The drugs that were most frequently combined with S-1 were oxaliplatin, irinotecan, and VEGF-inhibitors (bevacizumab or aflibercept), in line with current guidelines, or EGFR-inhibitors (panitumumab or cetuximab) that were used for conversion in selected patients but are not recommended in current guidelines [1, 21, 23]. The frequencies of AEs for S-1 as a single agent [16, 24–26] or in combination with oxaliplatin or irinotecan ± biologics are in line with previously published data [21, 24, 27–35]. The rates of AEs in our Safety cohort were similar to the rates observed in the SmPC cohort previously shared with EMA and no new risk-benefit concerns were raised.

In this analysis, the mPFS for first-line S-1 treatment in patients with mCRC was 11.0 months and mOS was 26.6 months. This is in line with previously published data with 4.4–8.4 months and 11.1–16.8 months for mPFS and mOS, respectively, for FP monotherapy, and 5.1–12.2 months and 14.5–29.7 months, respectively, for combinations with oxaliplatin or irinotecan [16, 24–35].

The mPFS in later-line treatment was 2.8 months and mOS was 12.7 months, in line with 1.9–5.7 months and 6.4–11.2 months, for mPFS and mOS, respectively, for other regimens [36–41].

There are very few alternatives or strategies to continue effective treatment after FP-induced cardiotoxicity in mCRC. In the curative-intent setting, there are no options other than rechallenge with CAP/i.v. 5-FU with cardioprotective treatment under telemetry guided by a cardio-oncologist, or switch to an alternative FP such as S-1, as all data in the neoadjuvant/adjuvant situation are based on FPs, either as single agent or combined with oxaliplatin [1, 42]. However, rechallenge with CAP or infused 5-FU puts the patient at high risk of cardiotoxicity recurrence and even mortality [3, 43]. The effect of dose reduction with or without prophylactic cardioprotective drugs is modest at best [5, 44, 45]. Three case series of 5, 6, and 10 patients found that bolus 5-FU is feasible after previous cardiotoxicity on infused/oral FP with recurrence of cardiotoxicity in 0%–20% [13, 46, 47]. Raltitrexed has been reported to be an option after FP-induced cardiotoxicity in two small retrospective studies with 25 and 32 mCRC patients [48, 49]. However, raltitrexed is not widely used due to modest activity, a difficult toxicity profile, and relatively high mortality [50, 51]. The only alternative in the conversion setting is to continue with an FP, of which S-1 is easy to use and apparently safe. In combination with oxaliplatin or irinotecan and a biologic, it yields high response rates and thus maximizes the chance for cure [1, 52].

Unfortunately for some patients, the most effective approach is not feasible. Many elderly or unfit patients need a more de-escalated chemotherapeutic strategy approach [1]. FPs are still the best choice here, providing meaningful and durable responses with emphasis on maintaining quality of life [25]. Also, for this group, S-1 can provide a safe and efficacious treatment option [16, 26].

The benefits of metastasectomy and/or LAT are well documented, and the goal should be to achieve resectability in as many cases as possible, as a preserved curative intent of treatment is an important factor in pursuing better OS [1, 53], as seen in these patients. In this study, up to half of the patients underwent curative intent metastasectomy and/or LAT, of which 64% had the procedure after switching to S-1-based chemotherapy. We observed no safety concerns regarding S-1 and metastatic surgery for either liver, lung, or cytoreductive surgery. As cardiotoxicity almost always leads to discontinuation of FP, being able to continue effective combination chemotherapy with S-1 is extremely important for enabling conversion to resectability. In this analysis, we show this strategy to be feasible with a success rate of 95% for patients who were able to undergo effective conversion therapy with SOX or IRIS ± biologics.

This substudy in mCRC patients has limitations due to its retrospective nature [13]. It is likely that a prospective setting would have provided more comprehensive data, but inclusion time would be an issue. Also, a randomized study would be unethical as the experimental arm would include rechallenge of FP under cardioprotection or raltitrexed, with unacceptably high mortality risks.

In summary, this study confirms that switching to S-1-based therapy is a feasible and safe option for patients with mCRC when conventional FPs are discontinued due to cardiotoxicity. Furthermore, S-1 can be safely combined with several chemotherapeutic and biologic drugs, retaining the full benefits of standard FP combination treatment options in this setting. Moreover, S-1-based therapy allows patients with mCRC to continue and complete effective treatment and to go through surgical metastasectomy without added risk.

## Supplementary Material

Detailed analysis of metastatic colorectal cancer patients who developed cardiotoxicity on another fluoropyrimidine and switched to S-1 treatment (subgroup analysis of the CardioSwitch-study)

Detailed analysis of metastatic colorectal cancer patients who developed cardiotoxicity on another fluoropyrimidine and switched to S-1 treatment (subgroup analysis of the CardioSwitch-study)

## Data Availability

The data collected for this study can be made available to others in de-identified form after all primary and secondary endpoints have been published, in the presence of a data transfer agreement, and if the purpose of use complies with Finnish and European legislation. Requests for data sharing can be made to the corresponding author, including a proposal that must be approved by the steering committee.

## References

[1] Cervantes A, Adam R, Roselló S, et al. Metastatic colorectal cancer: ESMO Clinical Practice Guideline for diagnosis, treatment and follow-up. Ann Oncol. 2023;34(1);10–32. 36307056 10.1016/j.annonc.2022.10.003

[2] Polk A, Vaage-Nilsen M, Vistisen K, Nielsen DL. Cardiotoxicity in cancer patients treated with 5-fluorouracil or capecitabine: a systematic review of incidence, manifestations and predisposing factors. Cancer Treat Rev. 2013;39:974–84. 10.1016/j.ctrv.2013.03.005 23582737

[3] Sorrentino MF, Kim J, Foderaro AE, Truesdell AG. 5-fluorouracil induced cardiotoxicity: review of the literature. Cardiol J. 2012;19:453–8. 10.5603/CJ.2012.0084 23042307

[4] Polk A, Vistisen K, Vaage-Nilsen M, Nielsen DL. A systematic review of the pathophysiology of 5-fluorouracil-induced cardiotoxicity. BMC Pharmacol Toxicol. 2014;15:47. 10.1186/2050-6511-15-47 25186061 PMC4170068

[5] Dyhl-Polk A, Vaage-Nilsen M, Schou M, Vistisen KK, Lund CM, Kümler T. Incidence and risk markers of 5-fluorouracil and capecitabine cardiotoxicity in patients with colorectal cancer. Acta Oncol. 2020;59:475–83. 10.1080/0284186X.2019.1711164 31931649

[6] Kwakman JJ, Simkens LH, Mol L, Kok WE, Koopman M, Punt CJ. Incidence of capecitabine-related cardiotoxicity in different treatment schedules of metastatic colorectal cancer: a retrospective analysis of the CAIRO studies of the Dutch colorectal cancer group. Eur J Cancer. 2017;76:93–99. 10.1016/j.ejca.2017.02.009 28286287

[7] Jurczyk M, Król M, Midro A, Kurnik-Łucka M, Poniatowski A, Gil K. Cardiotoxicity of fluoropyrimidines: Epidemiology, mechanisms, diagnosis, and management. J Clin Med. 2021;10:4426. 10.3390/jcm10194426 34640443 PMC8509845

[8] Lu Y, Deng S, Dou Q, et al. Treatment-related coronary disorders of fluoropyrimidine administration: a systematic review and meta-analysis. Front Pharmacol. 2022;13:885699. 10.3389/fphar.2022.885699 35645806 PMC9140752

[9] Deboever G, Hiltrop N, Cool M, Lambrecht G. Alternative treatment options in colorectal cancer patients with 5-fluorouracil- or capecitabine-induced cardiotoxicity. Clin Colorectal Cancer. 2013;12:8–14. 10.1016/j.clcc.2012.09.003 23102544

[10] Ajani JA, Rodriguez W, Bodoky G, et al. Multicenter phase III comparison of cisplatin/S-1 with cisplatin/infusional fluorouracil in advanced gastric or gastroesophageal adenocarcinoma study: the FLAGS trial. J Clin Oncol. 2010;28:1547–53. 10.1200/JCO.2009.25.4706 20159816

[11] Ter Veer E, Ngai LL, Valkenhoef GV, et al. Capecitabine, 5-fluorouracil and S-1-based regimens for previously untreated advanced oesophagogastric cancer: a network meta-analysis. Sci Rep. 2017;7:7142. 10.1038/s41598-017-07750-3 28769123 PMC5541083

[12] Kwakman JJM, Baars A, van Zweeden AA, de Mol P, Koopman M, Kok WEM, Punt CJA. Case series of patients treated with the oral fluoropyrimidine S-1 after capecitabine-induced coronary artery vasospasm. Eur J Cancer. 2017;81:130–4. 10.1016/j.ejca.2017.05.022 28623776

[13] Osterlund P, Kinos S, Pfeiffer P, et al. Continuation of fluoropyrimidine treatment with S-1 after cardiotoxicity on capecitabine- or 5-fluorouracil-based therapy in patients with solid tumours: a multicentre retrospective observational cohort study. ESMO Open. 2022;7:100427. 10.1016/j.esmoop.2022.100427 35798468 PMC9291631

[14] Punt CJA, Kwakman JJM, Mol L, PLCRC working group. Long-term safety data on S-1 administered after previous intolerance to capecitabine-containing systemic treatment for metastatic colorectal cancer. Clin Colorectal Cancer. 2022;21:229–35. 10.1016/j.clcc.2022.02.004 35341693

[15] Derksen JWG, Smit KC, May AM, Punt CJA. Systematic review and non-inferiority meta-analysis of randomised phase II/III trials on S-1-based therapy versus 5-fluorouracil- or capecitabine-based therapy in the treatment of patients with metastatic colorectal cancer. Eur J Cancer. 2022;166:73–86. 10.1016/j.ejca.2022.02.004 35279472

[16] Kwakman JJM, Simkens LHJ, van Rooijen JM, et al. Randomized phase III trial of S-1 versus capecitabine in the first-line treatment of metastatic colorectal cancer: SALTO study by the Dutch colorectal cancer group. Ann Oncol. 2017;28:1288–93. 10.1093/annonc/mdx122 28383633

[17] European Medicines Agency. EMA/CHMP/714788/2021. Summary of positive opinion [Internet]. [cited 12-08-2023]. Available from: https://www.ema.europa.eu/en/documents/smop/chmp-post-authorisation-summary-positive-opinion-teysuno-ii-45_en.pdf

[18] Teysuno (S-1) summary of product characteristics [Internet]. [cited 12-08-2023]. Available from: https://www.ema.europa.eu/en/medicines/human/EPAR/teysuno

[19] Glimelius B. Biochemical modulation of 5-fluorouracil: a randomized comparison of sequential methotrexate, 5-fluorouracil and leucovorin versus sequential 5-fluorouracil and leucovorin in patients with advanced symptomatic colorectal cancer. The Nordic Gastrointestinal Tumor Adjuvant Therapy Group. Ann Oncol. 1993;4:235–40. 10.1093/oxfordjournals.annonc.a058463 8471555

[20] Sørbye H, Dahl O. Nordic 5-fluorouracil/leucovorin bolus schedule combined with oxaliplatin (Nordic FLOX) as first-line treatment of metastatic colorectal cancer. Acta Oncol. 2003;42:827–31. 10.1080/02841860310018972 14968943

[21] Guren TK, Thomsen M, Kure EH, et al. Cetuximab in treatment of metastatic colorectal cancer: final survival analyses and extended RAS data from the NORDIC-VII study. Br J Cancer. 2017;116:1271–8. 10.1038/bjc.2017.93 28399112 PMC5482736

[22] Glimelius B, Sørbye H, Balteskard L, et al. A randomized phase III multicenter trial comparing irinotecan in combination with the Nordic bolus 5-FU and folinic acid schedule or the bolus/infused de Gramont schedule (Lv5FU2) in patients with metastatic colorectal cancer. Ann Oncol. 2008;19:909–14. 10.1093/annonc/mdm588 18209013

[23] Maughan TS, Adams RA, Smith CG, et al. Addition of cetuximab to oxaliplatin-based first-line combination chemotherapy for treatment of advanced colorectal cancer: results of the randomised phase 3 MRC COIN trial. Lancet. 2011;377:2103–14. 10.1016/S0140-6736(11)60613-2 21641636 PMC3159415

[24] Douillard JY, Cunningham D, Roth AD, et al. Irinotecan combined with fluorouracil compared with fluorouracil alone as first-line treatment for metastatic colorectal cancer: a multicentre randomised trial. Lancet. 2000;355:1041–7. 10.1016/S0140-6736(00)02034-1 10744089

[25] Cunningham D, Lang I, Marcuello E, et al. Bevacizumab plus capecitabine versus capecitabine alone in elderly patients with previously untreated metastatic colorectal cancer (AVEX): an open-label, randomised phase 3 trial. Lancet Oncol. 2013;14:1077–85. 10.1016/S1470-2045(13)70154-2 24028813

[26] Winther SB, Liposits G, Skuladottir H, et al. Reduced-dose combination chemotherapy (S-1 plus oxaliplatin) versus full-dose monotherapy (S-1) in older vulnerable patients with metastatic colorectal cancer (NORDIC9): a randomised, open-label phase 2 trial. Lancet Gastroenterol Hepatol. 2019;4:376–88. 10.1016/S2468-1253(19)30041-X 30852136

[27] de Gramont A, Figer A, Seymour M, et al. Leucovorin and fluorouracil with or without oxaliplatin as first-line treatment in advanced colorectal cancer. J Clin Oncol. 2000;18:2938–47. 10.1200/JCO.2000.18.16.2938 10944126

[28] Cassidy J, Clarke S, Díaz-Rubio E, et al. Randomized phase III study of capecitabine plus oxaliplatin compared with fluorouracil/folinic acid plus oxaliplatin as first-line therapy for metastatic colorectal cancer. J Clin Oncol. 2008;26:2006–12. 10.1200/JCO.2007.14.9898 18421053

[29] Saltz LB, Clarke S, Díaz-Rubio E, et al. Bevacizumab in combination with oxaliplatin-based chemotherapy as first-line therapy in metastatic colorectal cancer: a randomized phase III study. J Clin Oncol. 2008;26:2013–19. 10.1200/JCO.2007.14.9930 18421054

[30] Hurwitz H, Fehrenbacher L, Novotny W, et al. Bevacizumab plus irinotecan, fluorouracil, and leucovorin for metastatic colorectal cancer. N Engl J Med. 2004;350:2335–42. 10.1056/NEJMoa032691 15175435

[31] Nishizawa Y, Haraguchi N, Kim H, et al. Randomized phase II study of SOX+B-mab versus SOX+C-mab in patients with previously untreated recurrent advanced colorectal cancer with wild-type KRAS (MCSGO-1107 study). BMC Cancer. 2021;21:947. 10.1186/s12885-021-08690-y 34425776 PMC8381542

[32] Baba H, Yamada Y, Takahari D, et al. S-1 and oxaliplatin (SOX) plus bevacizumab versus mFOLFOX6 plus bevacizumab as first-line treatment for patients with metastatic colorectal cancer: updated overall survival analyses of the open-label, non-inferiority, randomised phase III: SOFT study. ESMO Open. 2017;2:e000135. 10.1136/esmoopen-2016-000135 28761727 PMC5519807

[33] Muro K, Boku N, Shimada Y, et al. Irinotecan plus S-1 (IRIS) versus fluorouracil and folinic acid plus irinotecan (FOLFIRI) as second-line chemotherapy for metastatic colorectal cancer: a randomised phase 2/3 non-inferiority study (FIRIS study). Lancet Oncol. 2010;11:853–60. 10.1016/S1470-2045(10)70181-9 20708966

[34] Sadahiro S, Suzuki T, Okada K, et al. Oral S-1 with 24-h infusion of irinotecan plus Bevacizumab versus FOLFIRI plus Bevacizumab as first-line chemotherapy for metastatic colorectal cancer: an open-label randomized Phase II trial. Oncology. 2020;98:637–42. 10.1159/000507293 32474564 PMC7592907

[35] Yamada Y, Denda T, Gamoh M, et al. S-1 and irinotecan plus bevacizumab versus mFOLFOX6 or CapeOX plus bevacizumab as first-line treatment in patients with metastatic colorectal cancer (TRICOLORE): a randomized, open-label, phase III, noninferiority trial. Ann Oncol. 2018;29:624–31. 10.1093/annonc/mdx816 29293874 PMC5889030

[36] Yoshino T, Mizunuma N, Yamazaki K, et al. TAS-102 monotherapy for pretreated metastatic colorectal cancer: a double-blind, randomised, placebo-controlled phase 2 trial. Lancet Oncol. 2012;13:993–1001. 10.1016/S1470-2045(12)70345-5 22951287

[37] Mayer RJ, Van Cutsem E, Falcone A, et al. Randomized trial of TAS-102 for refractory metastatic colorectal cancer. N Engl J Med. 2015;372:1909–19. 10.1056/NEJMoa1414325 25970050

[38] Grothey A, Van Cutsem E, Sobrero A, et al. Regorafenib monotherapy for previously treated metastatic colorectal cancer (CORRECT): an international, multicentre, randomised, placebo-controlled, phase 3 trial. Lancet. 2013;381:303–12. 10.1016/S0140-6736(12)61900-X 23177514

[39] Li J, Qin S, Xu R, et al. Regorafenib plus best supportive care versus placebo plus best supportive care in Asian patients with previously treated metastatic colorectal cancer (CONCUR): a randomised, double-blind, placebo-controlled, phase 3 trial. Lancet Oncol. 2015;16:619–29. 10.1016/S1470-2045(15)70156-7 25981818

[40] Arnold D, Prager GW, Quintela A, et al. Beyond second-line therapy in patients with metastatic colorectal cancer: a systematic review. Ann Oncol. 2018;29:835–56. 10.1093/annonc/mdy038 29452346 PMC5913602

[41] Bennouna J, Sastre J, Arnold D, et al. Continuation of bevacizumab after first progression in metastatic colorectal cancer (ML18147): a randomised phase 3 trial. Lancet Oncol. 2013;14:29–37. 10.1016/S1470-2045(12)70477-1 23168366

[42] Argilés G, Tabernero J, Labianca R, et al. Localised colon cancer: ESMO clinical practice guidelines for diagnosis, treatment and follow-up. Ann Oncol. 2020;31:1291–305. 10.1016/j.annonc.2020.06.022 32702383

[43] Saif MW, Shah MM, Shah AR. Fluoropyrimidine-associated cardiotoxicity: revisited. Expert Opin Drug Saf. 2009;8:191–202. 10.1517/14740330902733961 19309247

[44] Jensen SA, Sørensen JB. Risk factors and prevention of cardiotoxicity induced by 5-fluorouracil or capecitabine. Cancer Chemother Pharmacol. 2006;58:487–93. 10.1007/s00280-005-0178-1 16418875

[45] Punt CJA, Heinemann V, Maughan T, et al. Fluoropyrimidine-induced hand-foot syndrome and cardiotoxicity: recommendations for the use of the oral fluoropyrimidine S-1 in metastatic colorectal cancer. ESMO Open. 2023;8:101199. 10.1016/j.esmoop.2023.101199 37018874 PMC10163153

[46] Saif MW, Garcon MC, Rodriguez G, Rodriguez T. Bolus 5-fluorouracil as an alternative in patients with cardiotoxicity associated with infusion 5-fluorouracil and capecitabine: a case series. In Vivo. 2013;27:531–4. 23812226

[47] Chakrabarti S, Sara J, Lobo R, et al. Bolus 5-fluorouracil (5-FU) in combination with oxaliplatin is safe and well tolerated in patients who experienced coronary vasospasm with infusional 5-FU or capecitabine. Clin Colorectal Cancer. 2019;18:52–7. 10.1016/j.clcc.2018.09.006 30396850

[48] Ransom D, Wilson K, Fournier M, et al. Final results of Australasian Gastrointestinal Trials Group ARCTIC study: an audit of raltitrexed for patients with cardiac toxicity induced by fluoropyrimidines. Ann Oncol. 2014;25:117–21. 10.1093/annonc/mdt479 24299960

[49] Batra A, Rigo R, Hannouf MB, Cheung WY. Real-world safety and efficacy of raltitrexed in patients with metastatic colorectal cancer. Clin Colorectal Cancer. 2021;20:e75–81. 10.1016/j.clcc.2020.09.006 33268287

[50] Hind D, Tappenden P, Tumur I, Eggington S, Sutcliffe P, Ryan A. The use of irinotecan, oxaliplatin and raltitrexed for the treatment of advanced colorectal cancer: systematic review and economic evaluation. Health Technol Assess. 2008;12:iii–162. 10.3310/hta12150 18462574

[51] Popov I, Carrato A, Sobrero A, et al. Raltitrexed (Tomudex) versus standard leucovorin-modulated bolus 5-fluorouracil: results from the randomised phase III Pan-European Trial in Adjuvant Colon Cancer 01 (PETACC-1). Eur J Cancer. 2008;44:2204–11. 10.1016/j.ejca.2008.07.002 18707870

[52] Folprecht G, Grothey A, Alberts S, Raab HR, Köhne CH. Neoadjuvant treatment of unresectable colorectal liver metastases: correlation between tumour response and resection rates. Ann Oncol. 2005;16:1311–9. 10.1093/annonc/mdi246 15870084

[53] Osterlund P, Salminen T, Soveri LM, et al. Repeated centralized multidisciplinary team assessment of resectability, clinical behavior, and outcomes in 1086 Finnish metastatic colorectal cancer patients (RAXO): a nationwide prospective intervention study. Lancet Reg Health Eur. 2021;3:100049. 10.1016/j.lanepe.2021.100049 34557799 PMC8454802

