# Supplementary material for: Detailed analysis of metastatic colorectal cancer patients who developed cardiotoxicity on another fluoropyrimidine and switched to S-1 treatment (subgroup analysis of the CardioSwitch-study)
Source: Acta Oncol. 2024 May 2;63:24023. doi: 10.2340/1651-226X.2024.24023 (PMC11332541; doi:10.2340/1651-226X.2024.24023)
Supplement: Detailed analysis of metastatic colorectal cancer patients who developed cardiotoxicity on another fluoropyrimidine and switched to S-1 treatment (subgroup analysis of the CardioSwitch-study) [file AO-63-24023-s1.pdf]

Supplementary material has been published as submitted. It has not been copyedited or typeset by Acta Oncologica.

Supplementary Table 1. Baseline characteristics for the SmPC cohort and the Efficacy cohort

**Characteristic**		**SmPC cohort, n=53 n (%)**	**Efficacy cohort, n=66 n (%)**
Age, years	Median (range)	68 (19-85)	68 (19-85)
Sex	Male	32 (60)	40 (61)
ECOG	0-1	45 (85)	57 (88)
	2	7 (13)	8 (12)
	Not available	1 (2)	1 (2)
Cardiovascular comorbidity	Yes	26 (49)	33 (50)
	No	27 (51)	33(50)
Primary tumor location	Right colon	16 (30)	21 (27)
	Left colon	20 (38)	26 (33)
	Rectum	8 (15)	15 (19)
	Colon unspecified	9 (17)	16 (21)
Primary tumor resected	Yes	38 (72)	46 (70)
	No	15 (28)	20 (30)
Pelvic radiotherapy	Yes	2 (4)	4 (6)
	No	51 (96)	62 (94)
Treatment intent of FP causing cardiotoxicity	Adjuvant-like*	14 (26)	22 (33)
	Neo-adjuvant/Conversion	17 (32)	21 (32)
	1st line	18 (34)	19 (29)
	2nd line	4 (8)	4 (6)

*After metastasectomy and/or local ablative therapy (LAT)
ECOG, Eastern Cooperative Oncology Group; FP, fluoropyrimidine

Supplementary Table 2. Baseline comorbidities for included patients

		Safety cohortN=78<br>n (%)
Cardiovascular		39 (50)
	Hypertension	29 (37)
	Ischemic heart disease	9 (12)
	Arrythmia	7 (9)
	Cardiac heart failure	3 (4)
	Cerebrovascular event	3 (4)
	Cardiomyopathy	2 (3)
	Valvular heart disease	2 (3)
	Atherosclerosis	1 (1)
	Arterial or venous thromboembolism	1 (1)
	RBBB, LBBB or prolonged QT	1 (1)
	Myocardial infarction	1 (1)
Metabolic		20 (26)
	Dyslipidemia	16 (21)
	T2DM	5 (6)
	Obesity	5 (6)
Renal		5 (6)
	Mild (CC 50-85ml/min)	3 (4)
	Normal (CC >85 ml/min)	2 (3)
Lung		6 (8)
	Chronic obstructive pulmonary disease	5 (6)
	Asthma	1 (1)
Other		27 (35)
	Hypothyroidism	6 (8)
	Other malignancy	5 (6)
	Gastro-esophageal reflux	4 (5)
	Benign prostate hyperplasia	4 (5)
	Musculoskeletal disorders	4 (5)
	Psychiatric disorders	4 (5)
	Skin disorders	2 (3)
	Glaucoma	1 (1)
	Gout	1 (1)
	Amyloidosis	1 (1)
	Inflammatory bowel disease	1 (1)
	Brain injury	1 (1)
	Celiac disease	1 (1)
	Migraine	1 (1)
	Polyneuropathy	1 (1)
	Sjogren’s syndrome	1 (1)

Supplementary Table 3. Initial cardiotoxicity for patients in the SmPC cohort and the Efficacy cohort.

		**SmPC, n=53 n (%)**	**Efficacy cohort, n=66 n (%)**
Time to cardiotoxicity from treatment onset, days	Median (range)	6 (0-466)	6 (0-466)
Cycles to cardiotoxicity	1	33 (62)	44 (67)
	2	10 (19)	10 (15)
	3	4 (8)	4 (6)
	4 to 14	6 (11)	8 (12)
Multiple cardiotoxic symptoms	No	42 (79)	55 (83)
	Yes	11 (21)	11 (17)
Cardiotoxicity	Acute coronary syndrome	22 (42)	27 (41)
	Atrial fibrillation	4 (7)	4 (6)
	Chest pain	30 (57)	38 (58)
	Heart failure	2 (4)	2 (3)
	Tachycardia	4 (8)	4 (6)
	Bradycardia	1 (2)	1 (2)
	Prolonged QT interval	1 (1)	1 (2)
Worst cardiotoxicity grade	1	7 (13)	8 (12)
	2	21 (40)	27 (41)
	3	21 (40)	27 (41)
	4	4 (8)	4 (6)
Action with fluoropyrimidine	Temporarily discontinued	3 (6)	3 (5)
	Permanently discontinued	50 (94)	63 (96)
	Recovery from cardiac event		
	With sequelae	1 (2)	1 (4)
	Without sequelae	52 (98)	65 (96)
Causality	Possibly related	3 (6)	6 (9)
	Probably related	34 (64)	44 (67)
	Related	16 (30)	16 (24)

Supplementary Table 4. Other adverse events for the SmPC cohort

	**Initial FP-based treatment, n=53 n (%)**	**After switch to S-1-based treatment, n=53, n (%)**
Neutropenia	-	4 (8)
Anemia	-	-
Stomatitis	1 (2)	1 (2)
Diarrhea	2 (4)	2 (4)
Nausea	1 (2)	1 (2)
Infection	-	4 (7)
Neuropathy	4 (8)	8 (15)
Hand-foot syndrome	1 (2)	1 (2)
Thromboembolism	-	3 (6)
Fatigue	-	1 (2)
Pneumonitis	-	1 (2)
Rash	-	1 (2)
Any grade 2-4 non-hematologic event	8 (15)	21 (36)

FP, fluoropyrimidine
