# Supplementary figures and images for: Detailed analysis of metastatic colorectal cancer patients who developed cardiotoxicity on another fluoropyrimidine and switched to S-1 treatment (subgroup analysis of the CardioSwitch-study)
Source: Acta Oncol. 2024 May 2;63:24023. doi: 10.2340/1651-226X.2024.24023 (PMC11332541; doi:10.2340/1651-226X.2024.24023)

## Disease status at cardiotoxicity event

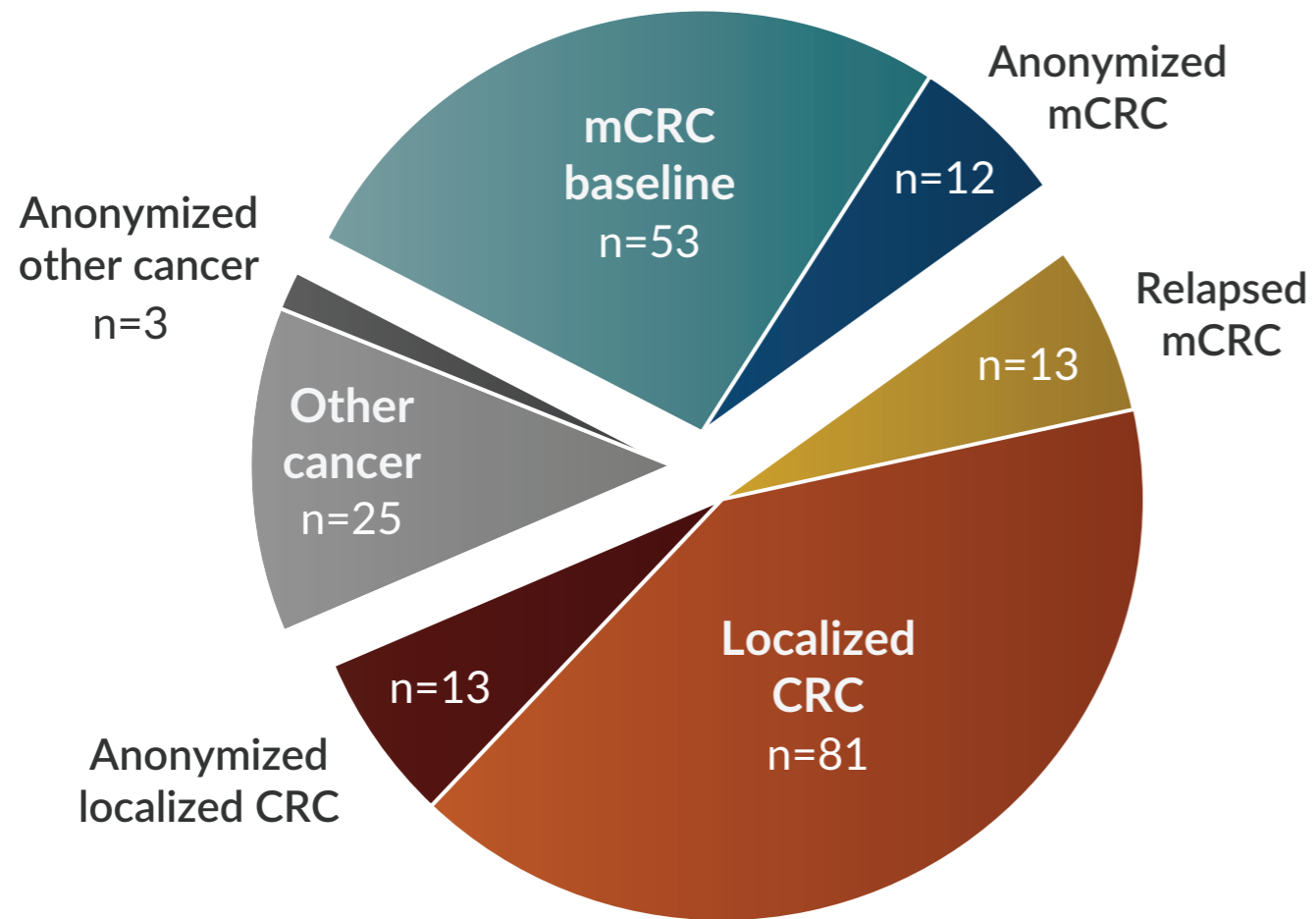

## S-1 treatment for mCRC

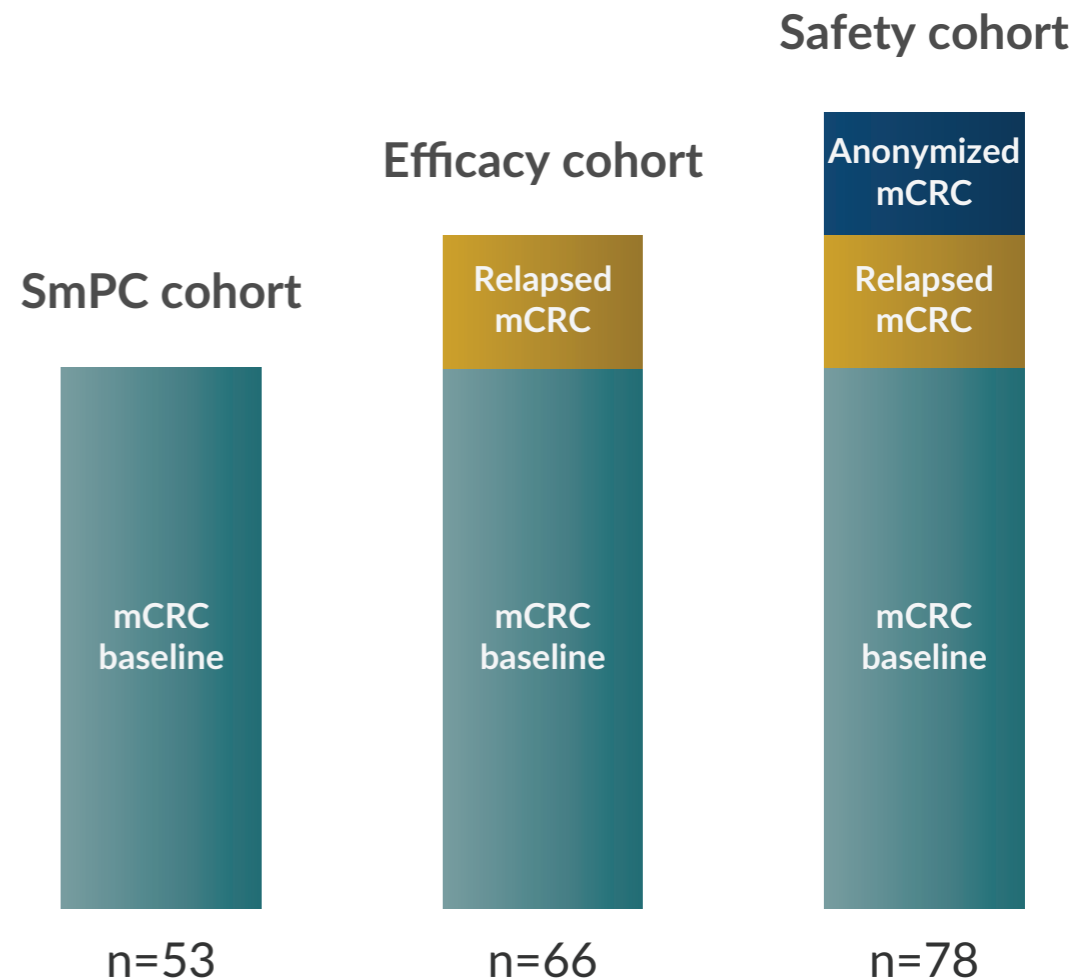

Supplement: Detailed analysis of metastatic colorectal cancer patients who developed cardiotoxicity on another fluoropyrimidine and switched to S-1 treatment (subgroup analysis of the CardioSwitch-study) [file AO-63-24023-s2.pdf]
